# Intracranial Pressure Monitoring, Heart Rate Variability, Baroreflex Sensitivity, and Signal Complexity During Neurointensive Care after Decompressive Craniectomy in Malignant Middle Cerebral Artery Infarction

**DOI:** 10.1007/s12028-026-02506-2

**Published:** 2026-04-07

**Authors:** Modar Alhamdan, Anders Hånell, Timothy Howells, Odin Joensen, Fartein Velle, Anders Lewén, Per Enblad, Teodor Svedung Wettervik

**Affiliations:** Department of Medical Sciences, Section of Neurosurgery, Uppsala University, 751 85 Uppsala, Sweden

**Keywords:** Heart rate variability, Baroreflex sensitivity, Signal complexity, Multiscale entropy, Malignant middle cerebral artery infarction, Neurointensive care

## Abstract

**Background:**

Malignant middle cerebral artery infarction (MMI) is generally treated with decompressive craniectomy (DC) followed by neurointensive care (NIC). However, prognostic values of autonomic and complexity indices remain unclear. We aimed to explore heart rate variability (HRV), baroreflex sensitivity (BRS), and signal complexity (SC) in patients with MMI during post-DC NIC in relation to demographics, stroke severity, NIC variables [intracranial pressure (ICP), cerebral perfusion pressure (CPP), and Pressure Reactivity index (PRx)], and outcome.

**Methods:**

This single-center, retrospective study analyzed 70 ICP-monitored, typically sedated, and mechanically ventilated patients with MMI, during NIC after DC (2008–2022). HRV, BRS, and SC [multiscale entropy (MSE)] of heart rate, mean arterial pressure (MAP), ICP, and amplitude of ICP (AMP) were analyzed for the first seven NIC days post-DC. Correlations (Spearman) were explored for HRV, BRS, and SC with demographics, stroke severity, and NIC variables. Associations (multivariable ordinal logistic regression) were evaluated for HRV, BRS, and SC variables in relation to six-month modified Rankin Scale (mRS). Combinations of SC and NIC variables were explored in relation to mRS in heatmaps.

**Results:**

Median age was 57 years and 79% were male. At 6 months, median mRS was 4, and 7% died. HRV and BRS were reduced post-DC, but not associated with demographics, stroke severity, NIC variables, and mRS. Higher SC correlated (Spearman) with lower burden of secondary insults (NIC variables beyond pathological thresholds). Higher SC of MAP, ICP, and AMP correlated independently with lower six-month mRS in multivariable ordinal logistic regression. Low SC combined with pathological NIC variables correlated particularly with worse outcome in heatmaps.

**Conclusions:**

Lower systemic and cerebral SC correlated with greater burden of secondary insults and worse long-term outcome in MMI post-DC. Low SC may indicate overloaded systemic and cerebral compensatory mechanisms, resulting in reduced tolerance for secondary insults. SC may aid prognostication and fine-tune NIC to identify and prevent autoregulatory insults.

**Supplementary Information:**

The online version contains supplementary material available at 10.1007/s12028-026-02506-2.

## Introduction

Malignant middle cerebral artery (MCA) infarctions (MMI) represent more than 5% of all strokes,^1^ where an occlusion in the proximal MCA causes ischemia, leading to space-occupying, cytotoxic brain edema and intracranial hypertension [1, 2]. Without treatment, these patients usually experience deterioration in consciousness, brain herniation, and mortality approaching 80% of cases [3, 4]. Decompressive craniectomy (DC) is the main treatment strategy to alleviate the mass effect in MMI [5, 6], and has been shown to improve survival rates and functional outcome. [3, 4]

Neurointensive care (NIC) following DC in MMI is essential for physiological optimization to reduce secondary brain injury and improve recovery [7]. However, there is limited evidence regarding the types of monitoring and the physiological treatment targets that should be pursued in this condition [5, 6]. Invasive monitoring of intracranial pressure (ICP) and cerebral perfusion pressure (CPP) is used in some centers but not universally, and appropriate ICP targets in the absence of an intact skull remain uncertain. In practice, management strategies have been extrapolated from other severe acute brain injuries, such as traumatic brain injury (TBI), where multimodal neuromonitoring during NIC is employed to guide therapy and improve outcome [8, 9]. To advance the field, we recently investigated the relationships between ICP, CPP, cerebrovascular autoregulation (PRx), and clinical outcome after DC for MMI as a first step toward a deeper understanding of these physiological dynamics and their prognostic value [7]. Herein, the next step involved in-depth analyses of systemic and cerebral physiological variables by studying autonomic nervous system (ANS) function and signal complexity (SC) in MMI.

Impaired ANS is common after severe brain injury conditions, such as TBI and ischemic or hemorrhagic stroke [10–16]. Reduced heart rate variability (HRV) and baroreflex sensitivity (BRS) have been suggested to reflect ANS dysfunction [10–13] and predict unfavorable outcome in several acute brain injury conditions [10–16]. The compromised ANS typically has reduced parasympathetic tone and increased sympathetic activity [10, 17]. HRV describes the balance between sympathetic and parasympathetic activity through beat-to-beat (BtB) fluctuations in interbeat intervals (IBI, i.e., R-to-R intervals) [10–13, 17–19], while BRS captures BtB variations of IBI in relation to blood pressure [10–13, 17, 20–22]. Furthermore, physiological signals may contain patterns that describe physiological responses to internal and external factors, which can be indicated through SC. More complex signals reflect a larger physiological compensatory capacity for both spontaneous variations and pathological insults [23–26], which can be expressed with multiscale entropy (MSE) [23–27]. SC can be viewed as an even more multifaceted analysis of signal variability that captures the complex nonlinear and reactive behavior of physiological signals compared with raw variability indices of HRV and BRS [23–27]. Additionally, SC has been reported to robustly predict outcome in other brain injury conditions, such as TBI and aneurysmal subarachnoidal hemorrhage (aSAH) [23–26, 28–33]. The disrupted HRV, BRS, and SC have been suggested to correlate with a reduced physiological compensatory capacity and a heavier insult burden of key NIC cerebral variables (ICP, CPP, and PRx) [23–26, 28–31]. However, the dynamics and roles of HRV, BRS, and SC remain unexplored in MMI following DC. Thus, understanding these dynamics may improve prognostication of MMI patients after DC and aid NIC management of cerebral variables.

The primary hypothesis was that reduced SC of systemic and cerebral physiology would be associated with worse clinical outcome and a heavier insult burden of ICP, CPP and PRx. The aim of this study was to explore in depth the physiological variables reflecting ANS (HRV and BRS) and SC following MMI requiring DC. Specifically, we aimed to examine their relationships with demographic factors, stroke severity, and key NIC treatment targets (ICP, CPP, and PRx), as well as their potential prognostic value. We hypothesized that ANS and SC variables would be impaired and associated with greater stroke severity, disturbed cerebral physiology, and unfavorable outcome.

## Materials and Methods

### Patients and Study Design

A total of 101 adult patients with MMI, treated with DC from 2008 to 2022 at the Department of Neurosurgery, Uppsala University Hospital (Sweden), were considered for this observational, single-center study. Patients were ICP monitored at NIC, and typically sedated and mechanically ventilated. After exclusion of 11 patients with no ICP-monitoring, 14 patients with ICP-monitoring for less than 24 h, and 6 patients with missing outcome data, the final study cohort consisted of 70 patients (Supplementary Fig. 1).

The catchment area of the Department of Neurosurgery is about 2 million people distributed among eight counties in the middle of Sweden. In these counties, healthcare is provided through local hospitals, whereas advanced neurological and neurosurgical care is performed at the Uppsala University Hospital after referral from local hospitals. In the context of MMI, regardless of infarct laterality, patients who were relatively healthy pre-stroke, younger than ≈70 years, and exhibiting manifest/risk of neurological deterioration or brain herniation, were considered for DC and transported to Uppsala for clinical care at the neurointensive/intermediate care units, as described below.

### Management Protocol

The management of patients with MMI at the Department of Neurosurgery in Uppsala University Hospital has been described in detail in a previous study [7]. In short, indications for DC were reduced consciousness below 12 on the Glasgow Coma Scale (GCS) and significant radiological mass effect with over 5 mm midline shift. Patients who were conscious before DC (GCS 9–12) were typically extubated postoperatively, whereas patients with impaired consciousness before DC (GCS below 9) were kept intubated and mechanically ventilated after surgery. In the latter group, ICP was monitored using a parenchymal pressure device (Codman ICP Micro-Sensor; Codman & Shurtleff; Raynham; MA, USA). Neurointensive care targets were: ICP < 20 mm Hg, CPP > 60 mm Hg, pO_2_ > 12 kPa (90 mm Hg), pCO_2_ 4.5–5.5 kPa (34–41 mm Hg), and arterial glucose 5–10 mmol/L. Furthermore, a near-zero fluid balance, normal electrolytes, and normothermia were targeted. Basal strategies to control ICP included elevation of the head of the bed, mild hyperventilation and increased sedation/analgesia using propofol and morphine when needed. Drainage of cerebrospinal fluid through an external ventricular drain and thiopental infusion were used in rare refractory cases. Low CPP was treated mainly with intravenous (IV) colloid fluids and otherwise with inotropes/vasopressors. Neurological wake-up tests were performed around 3–6 times/day, but the patients were kept continuously sedated in case of elevated ICP.

### Data Collection

Medical records were reviewed retrospectively to collect clinical data. Age (years) and sex (male/female) were recorded. Intravenous thrombolysis (yes/no) or endovascular thrombectomy (yes/no) were noted. At admission to NIC, hemiparesis (yes/no), dysphasia (yes/no), infarct lateralization (right/left), and Glasgow Coma Scale motor score (GCS M) (scale) were documented. Immediately before DC, GCS M (scale) and pupillary reactivity (reactive/1 unreactive/2 unreactive) were noted. Midline shift (mm) and compression of basal cisterns (open/compressed/obliterated) were recorded both before and after DC. The infarct volume (cm^3^) was retrospectively assessed using BrainLab (Germany Headquarters, Munich, Germany) on the basis of the delimitable extent of the infarction on computed tomography (CT) scans pre-DC by one of the authors (TSW). Time from stroke onset to DC (h) was documented. Time under mechanical ventilation post-DC was presented as percentage of the first 7 days of NIC. The primary outcome measure was modified Rankin Scale (mRS) assessed at the nearest possible point to 6 months postoperatively on the basis of all available medical records. The timepoint for outcome assessment (months) was documented. The mRS ranges from 0 (no symptoms) to 6 (death) [34]. Favorable outcome was defined as mRS 0–3, while unfavorable 4–6.

All data processing was performed in RStudio (version 2025.09.0). Electrocardiography (ECG), arterial blood pressure (ABP), and ICP data were analyzed for the first 7 days (168 h) post-DC, where data were collected at a frequency of 100 Hz or higher using the Odin software from the NIC monitoring network [35]. ECG was monitored using a 3-lead system and ABP using an arterial line, with reference point at heart level. Heartbeats were detected through the ECG waveform values using discrete wavelet transform with Daubechies-4 wavelet at four decomposition levels [36, 37]. Local maxima were detected in the second-level detail coefficient and corresponding R peaks were marked in the original ECG waveform. Timestamps for unique peaks were obtained, and IBIs (i.e., RR intervals) were calculated in seconds. BtB HR [beats per minute (BPM)] was then computed as 60 (s) divided by each IBI (s). BtB systolic blood pressure (SBP) was computed as the highest ABP value between two consecutive R peaks detected in the ECG waveform. Likewise, the BtB pulse amplitude of ICP (AMP) was defined as the difference between the highest and lowest values of the ICP waveform located between two consecutive R-peaks [38]. CPP was defined as the difference between MAP and ICP [39]. PRx was determined as the moving Pearson’s correlation between 10-s averages of ICP and ABP over a moving 5-min window [40–42]. Similarly, pulse amplitude index (PAx) was calculated as the moving Pearson’s correlation between 10-s averages of AMP and MAP over a moving 5-min window [41]. Valid monitoring time (VMT) was defined as monitoring time after exclusion of artifacts and monitoring gaps with unavailable data. The percentage of VMT (%VMT) of key NIC targets beyond certain thresholds were calculated to show insult burden. In accordance with our treatment targets, %VMT of ICP exceeding 20 mm Hg and CPP below 60 mm Hg were calculated and considered as insults. Additionally, %VMT of PRx surpassing +0.20 was calculated, since PRx values in this range have been suggested to indicate the limit of cerebral autoregulation in other brain injury conditions [43]. All physiologically implausible signals in all variables were automatically excluded as artifacts. Physiologically implausible data refer to observations beyond certain ranges for each variable: IBI 0.25–3.0 s corresponding to HR 20–240 BPM, SBP 50–250 mm Hg, and MAP 30–200 mm Hg. ICP values outside the range of 0–30 mm Hg were considered artifacts as well, since ICP rarely exceeds 30 mm Hg after DC.

### Data Analysis

#### Heart Rate Variability

HRV analyses were done in time-domain for the first 7 days of NIC post-DC in accordance with the standard measurements of the Task Force of the European Society of Cardiology and the North American Society of Pacing and Electrophysiology [18, 19, 44]. The computed variables were SDNN [standard deviation of all IBIs in (ms)] and RMSSD [root mean square of successive differences between adjacent IBIs (ms)]. Only SDNN and RMSSD were presented, since they provide an overview of HRV; SDNN reflects the overall HRV and is affected by both sympathetic and parasympathetic vagal activity, whereas RMSSD describes BtB HRV and is mainly affected by parasympathetic tone [19]. All data analyses were performed in RStudio (version 2025.09.0).

#### Baroreflex Sensitivity

BRS was determined using the sequential BRS method for the first 7 days of NIC following DC [20–22]. Each BtB SBP was paired with the immediately preceding IBI interval covering the same cardiac cycle, i.e., 0-beat delay. Non-overlapping sequences with at least three adjacent beats, where both SBP and IBI increase or decrease, were detected. Pearson’s correlation between SBP and IBI was then computed for all sequences. A sequence was valid if Pearson’s correlation was statistically significant (*p* < 0.05) and SBP variation exceeded 2.5 mm Hg. No minimum variation of IBI was required. BRS (ms/mm Hg) for each sequence was defined as the slope of the regression line for that sequence. Patient BRS was defined as median BRS of all sequence BRS values.

#### Multiscale Entropy

MSE is a tool that indicates the SC in a certain variable on the basis of several time scales [23–27]. MSE was analyzed for the first 7 days of NIC after DC, in accordance with established MSE methods [23–27], to calculate MSE complexity index (MSE-Ci) for HR, MAP, ICP, and AMP. Raw data of each variable were averaged in non-overlapping 10-s windows (scale 1). Every two adjacent values of scale 1 were averaged, resulting in a new data series (scale 2). Likewise, each three adjacent values of scale 1 were averaged into scale 3, and 20 timescales were similarly created for each variable. Sample entropy (SE) was computed for each time scale, employing an embedding dimension (*m*) of 2 and a tolerance coefficient (*r*) of 0.15 × standard deviation for each scale. SE was calculated as the negative logarithm of the conditional probability that two sequences of length (*m*) that are similar within a tolerance (*r*) remain similar at the next point. MSE-Ci was calculated as the area under the curve for the SE curve across all 20 timescales.

### Statistical Analyses

Categorical variables were described as numbers (percentages) and numerical variables were presented as medians (IQR). Spearman’s correlation test was employed to explore the association between SDNN, RMSSD, BRS, and MSE-Ci of HR, MAP, ICP, and AMP vs. demography, stroke severity, and key NIC targets (ICP, CPP, and PRx). The independent effects of physiological, HRV, BRS, and SC variables in relation to mRS were explored using multivariable proportional odds ordinal logistic regression analyses, adjusted for age (years), GCS M pre-DC (scale), infarct volume (cm^3^), and NIC time spent on mechanical ventilation (%). Odds ratios were presented per 1 unit. A *p*-value below 0.05 was considered statistically significant. Statistical analyses and visualizations were done in RStudio (version 2025.09.0).

### Data Visualization

Two-variable heatmaps were presented to demonstrate the interaction dynamics between the key NIC targets, ICP, CPP, and PRx, and SC of MAP, ICP, and AMP over the first 7 days of NIC following DC [7, 45]. Only SC variables (MAP, ICP, AMP) that correlated significantly to mRS were explored here, i.e., HRV, BRS, and HR SC were excluded. Median values for ICP, CPP, and PRx were calculated over non-overlapping 4-h windows, requiring a minimum of 75% VMT in each window. Similarly, SC was computed for MAP, ICP, and AMP over non-overlapping 4-h windows, requiring 75% of VMT in each window. The window of 4 h (3 h minimum) was chosen since SC calculations require at least 50 observations at the 20th timescale, i.e., 2.8 h of monitoring in the raw timescale [27]. Each ICP, CPP, and PRx was crossed with each SC of MAP, ICP, and AMP. Each variable range was divided into 5 bins, resulting in 25 grid cells (5 × 5) in each heatmap. Ranges were: ICP (0–20 mm Hg, 4 mm Hg/bin), CPP (50–100 mm Hg, 10 mm Hg/bin), PRx (−0.5 to 1.0, 0.3/bin), and all SC variables (0–30, 6/bin). All ranges were set depending on available observations to minimize the number of empty grid cells in the heatmaps. The %VMT (percentage) of the first 7 days of NIC was calculated for each grid cell for each patient. The %VMT was then correlated with mRS using Spearman’s correlation analysis, resulting in a *ρ*-value for each grid cell. The *ρ*-value was visualized employing a color scale, where correlation with better outcome was expressed as blue, and bad outcome as red. Grid cells containing data from fewer than five patients with at least 4 h of monitoring each were excluded (colored white). Finally, each grid cell was divided into nine equal subcells and Gaussian kernel blurring was applied, employing a standard deviation of four grid cells.

## Results

### Demographics and Baseline Characteristics

A total of 70 patients with MMI treated with DC from 2008 to 2022 were analyzed. Median age was 57 (IQR 51–62) years and most patients (79%) were male (Table 1). Intravenous thrombolysis had been administered in 30% of cases, while 10% had undergone endovascular thrombectomy. At admission, all patients presented with hemiparesis, 41% with dysphasia, and median GCS M was 6 (5–6). Most infarcts (59%) were lateralized to the right hemisphere and median infarct volume (cm^3^) was 254 (200–303), estimated on CT scans before DC. Preoperatively, median GCS M was 5 (5–6) and 17% exhibited impaired pupillary reactivity. In addition, prior to DC, median midline shift was 11 (8–13) mm, and basal cisterns were either compressed (87%) or obliterated (4%) in almost all cases. DC was performed at a median of 41 (29–60) h from stroke onset.

**Table 1:** Baseline characteristics and outcome

Variable	*N* (%) or median (IQR)
Patients, *n*	70 (100%)
Age (years)	57 (51–62)
Sex (male)	55 (79%)
Pre-stroke anticoagulants, *n* (%)	7 (10%)
Pre-stroke antiplatelets, *n* (%)	12 (17%)
GCS M at admission (scale)	6 (5–6)
Hemiparesis at admission (yes)	70 (100%)
Dysphasia at admission (yes)	29 (41%)
Intravenous thrombolysis (yes)	21 (30%)
Endovascular thrombectomy (yes)	7 (10%)
GCS M before DC (scale)	5 (5–6)
Infarct lateralization (left)	29 (41%)
Pupillary reactivity before DC (reactive/1 unreactive/2 unreactive)	58/12/0 (83/17/0%)
Midline shift pre-DC (mm)	11.0 (8.00–13.0)
Midline shift post-DC (mm)	3.00 (1.00–6.00)
Basal cisterns pre-DC (open/compressed/obliterated)	6/61/3 (9/87/4%)
Basal cisterns post-DC (open/compressed/obliterated)	59/9/2 (84/13/3%)
Infarct volume (cm^3^)	254 (200–303)
Time from stroke onset to DC (h)	41 (29–60)
DC area (cm^2^)	103 (92.0–118)
mRS (scale)	4 (4–4)
Favorable outcome (yes)	14 (20%)
Mortality (yes)	5 (7%)
Timepoint for outcome assessment (months)	10 (7–12)

*IQR* interquartile range, *GCS M* Glasgow Coma Scale Motor score, *DC* decompressive hemicraniectomy, *mRS* modified Rankin Scale. Favorable outcome 6-month mRS ≤ 3, mortality (at 6 months after DC)

### Physiological Characteristics During NIC

The monitoring time, extent of mechanical ventilation, and physiological characteristics are presented in Table 2. During the first 7 days of NIC following DC, the median available monitoring time was 158 h for ICP and 168 h for ECG and ABP, respectively. Patients were sedated and mechanically ventilated in a median of 100% (IQR 43–100%) of the corresponding monitoring time in the NIC unit. Median HR was 78 (69–88) BPM and median MAP was 89 (84–96) mm Hg. ICP had a median of 11 (9–13) mm Hg and exceeded 20 mm Hg in roughly 0.5% of VMT, while median AMP was 1.1 (0.80–1.7) mm Hg. Furthermore, the median CPP was 79 (74–85) mm Hg and %VMT of CPP below 60 mm Hg was 1.5%. Lastly, median PRx was 0.14 (0.076–0.28) and above 0.20 in approximately 44% of VMT, and median PAx was −0.11 (−0.17 to −0.050).

**Table 2:** Monitoring time, extent of mechanical ventilation, and physiological characteristics during NIC

Variable	Median (IQR)
Monitoring time of ICP (h)	158 (111–168)
Monitoring time of ECG (h)	168 (135–168)
Monitoring time of ABP (h)	168 (134–168)
Mechanical ventilation (%)	100 (42.9–100)
HR (BPM)	77.9 (69.3–87.6)
MAP (mm Hg)	88.9 (84.5–96.2)
ICP (mm Hg)	11.2 (9.08–13.0)
VMT ICP > 20 mm Hg (%)	0.500 (0.100–1.675)
AMP (mm Hg)	1.12 (0.800–1.67)
CPP (mm Hg)	79.1 (73.6–84.5)
VMT CPP < 60 mm Hg (%)	1.45 (0.525–4.48)
PRx	0.142 (0.0757–0.275)
PAx	−0.112 (−0.174 to −0.0497)
VMT PRx > 0.20 (%)	44.3 (35.3–57.7)

*NIC* neurointensive care, *IQR* interquartile range, *ICP* intracranial pressure, *ECG* electrocardiography, *ABP* arterial blood pressure, *mechanical ventilation (%)* percentage of post-DC NIC time spent on mechanical ventilation, *HR* heart rate, *BPM* beats per min, *MAP* mean arterial pressure, *VMT* monitoring time after exclusion of artifacts and monitoring gaps with unavailable data, *VMT ICP > 20 mm Hg (%)* percentage of valid monitoring time with ICP exceeding 20 mm Hg, *AMP* pulse amplitude of ICP, *CPP* cerebral perfusion pressure, *VMT CPP < 60 mm Hg (%)* percentage of valid monitoring time with CPP below 60 mm Hg, *PRx* pressure reactivity index, *PAx* pulse amplitude index, *VMT PRx > 0.20 (%)* percentage of valid monitoring time with PRx exceeding 0.20

### Heart Rate Variability, Baroreflex Sensitivity, and Multiscale Entropy

Median SDNN was 112 (IQR 92–142) ms, median RMSSD 29 (20–38) ms, and median BRS 3.6 (2.0–6.3) ms/mm Hg (Table 3). Median MSE-Ci was 10 (6.7–14) for HR, 14 (12–16) for MAP, 7.2 (4.8–9.4) for ICP, and 9.5 (6.8–14) for AMP.

**Table 3:** Heart rate variability, baroreflex sensitivity, and multiscale entropy

Variable	Median (IQR)
SDNN (ms)	112 (91.8–142)
RMSSD (ms)	28.5 (20.0–38.2)
BRS (ms/mm Hg)	3.57 (1.98–6.25)
HR MSE-Ci	9.97 (6.68–14.4)
MAP MSE-Ci	14.0 (12.0–16.2)
ICP MSE-Ci	7.22 (4.83–9.38)
AMP MSE-Ci	9.49 (6.77–14.2)

*IQR* interquartile range, *SDNN* standard deviation of all normal-to-normal RR intervals, *ms* milliseconds, *RMSSD* root mean square of adjacent RR interval differences, *BRS* sequence baroreflex sensitivity, *mm Hg* millimeters of mercury, *HR MSE-Ci* multiscale entropy complexity index of heart rate, *MAP MSE-Ci* multiscale entropy complexity index of mean arterial pressure, *ICP MSE-Ci* multiscale entropy complexity index of intracranial pressure, *AMP MSE-Ci* multiscale entropy complexity index of ICP pulse amplitude

### Correlation with Baseline Characteristics and NIC Targets

**Fig. 1:**
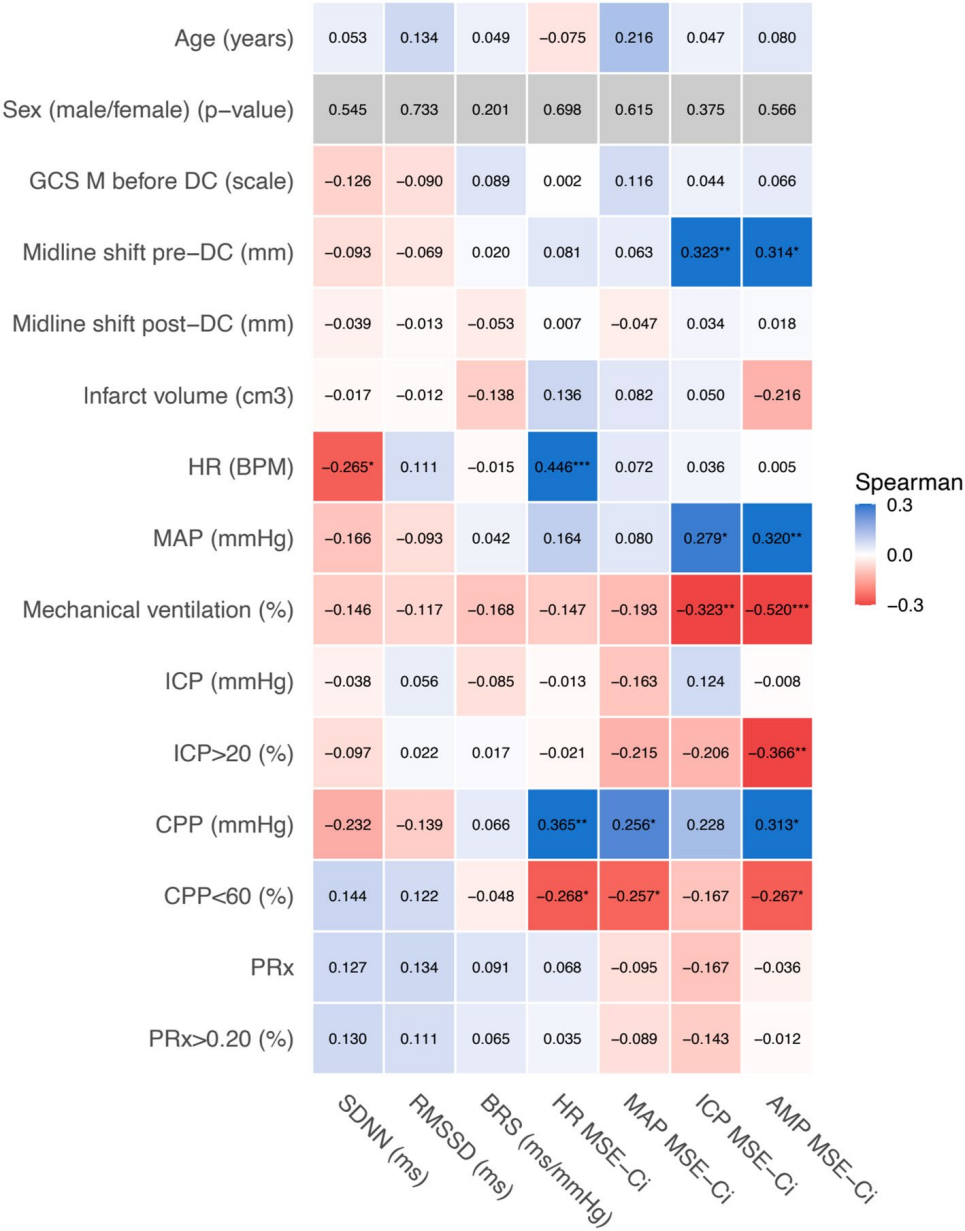
Spearman’s correlation: HRV, BRS, and SC vs. baseline characteristics and cerebral variables. * *p*-value < 0.05. ** *p*-value < 0.01. *** *p*-value < 0.001. Each grid cell contains the exact Spearman’s correlation coefficient (*ρ*). The color of the grid cell corresponds to *ρ*, where numerically positive correlation is expressed as blue and negative as red. Significance test for sex (male/female) was done using Mann–Whitney *U* test. *p*-Value below 0.05 was considered statistically significant. *HRV* heart rate variability, *BRS* baroreflex sensitivity, *SC* signal complexity, *GCS M* Glasgow Coma Scale motor score, *HR* heart rate, *BPM* beats per min, *MAP* mean arterial pressure, *mechanical ventilation (%)* percentage of post-DC NIC time spent on mechanical ventilation, *ICP*= intracranial pressure, *VMT* monitoring time after exclusion of artifacts and monitoring gaps with unavailable data, *VMT ICP > 20 mm Hg (%)* percentage of valid monitoring time with ICP exceeding 20 mm Hg, *VMT* valid monitoring time, *CPP* cerebral perfusion pressure, *VMT CPP < 60 mm Hg (%)* percentage of valid monitoring time with CPP below 60 mm Hg, *PRx* pressure reactivity index, *VMT PRx > 0.20 (%)* percentage of valid monitoring time with PRx exceeding 0.20, *SDNN* standard deviation of all normal-to-normal RR intervals, *ms* milliseconds, *RMSSD* root mean square of adjacent RR interval differences, *HR MSE-Ci* multiscale entropy complexity index of heart rate, *MAP MSE-Ci* multiscale entropy complexity index of mean arterial pressure, *ICP MSE-Ci* multiscale entropy complexity index of intracranial pressure, *AMP MSE-Ci* multiscale entropy complexity index of ICP pulse amplitude

Spearman’s analyses including baseline characteristics (Fig. 1) showed that greater midline shift pre-DC was associated with higher SC of ICP and AMP (*ρ* = 0.32; *p* < 0.01 and *ρ* = 0.31; *p* < 0.05, respectively), but there was otherwise no association between HRV, BRS, or SC variables and demography or stroke severity.

Several key NIC variables correlated significantly with HRV, BRS, and SC (Fig. 1). For instance, higher HR during NIC was associated with lower SDNN (*ρ* = −0.27; *p* < 0.05) and more complex HR signal (*ρ* 0.45; *p* < 0.001). Similarly, higher SC of ICP and AMP were associated with greater MAP during NIC (*ρ* = 0.28; *p* < 0.05 and *ρ* = 0.32; *p* < 0.01, respectively) and lower percentage of NIC time spent on mechanical ventilation (*ρ* = −0.32; *p* < 0.01 and *ρ* = −0.52; *p* < 0.001, respectively). Furthermore, higher CPP, lower %VMT of CPP below 60 mm Hg, and lower %VMT of ICP above 20 mm Hg correlated significantly to higher SC, but no significant links between cerebral variables and HRV or BRS were found. Specifically, higher CPP was linked to higher SC of HR (*ρ* = 0.37; *p* < 0.01), MAP (*ρ* 0.26; *p* < 0.05), and AMP (*ρ* = 0.25; *p* < 0.05). Likewise, lower %VMT spent with CPP below 60 mm Hg correlated with higher SC of HR (*ρ* = −0.27), MAP (*ρ* = −0.26), and AMP (*ρ* = −0.27; all *p* < 0.05). Lastly, higher %VMT of ICP exceeding 20 mm Hg was associated with lower AMP SC (*ρ* = −0.37; *p* < 0.01). Exact *p*-values are presented in Supplementary Table 1.

### Outcome

At 10 (7–12) months postoperatively, median mRS was 4 (4–4), 20% had recovered favorably, and the mortality rate was 7% (Table 1). In multivariable ordinal logistic regression analyses of mRS, adjusted for age (years), GCS M pre-DC (scale), infarct volume (cm^3^), and NIC time spent on mechanical ventilation (%), each physiological variable of interest was evaluated separately. In these models (Table 4), lower ICP (OR 1.2; *p* < 0.05), lower PRx (OR 103; *p* < 0.01), and lower PAx (OR 334; *p* < 0.05) were associated with lower mRS (favorable outcome). Additionally, higher SC of MAP (OR 0.73; *p* < 0.001), ICP (OR 0.73; *p* < 0.001), and AMP (OR 0.87; *p* < 0.01) correlated with lower mRS as well. Moreover, in subgroup analyses, patients with infarct lateralized to the right hemisphere demonstrated significantly better PRx and 6-month mRS (*p* < 0.05; Supplementary Table 2).

**Table 4:** Multivariable ordinal logistic regression: physiological variables vs. mRS

Variable	Odds ratio (95% confidence interval)	*p*-Value
HR (BPM)	1.01 (0.961–1.06)	0.769
MAP (mm Hg)	1.01 (0.950–1.07)	0.793
ICP (mm Hg)	***1.15 (1.04–1.30)***	***0.0218***
AMP (mm Hg)	0.885 (0.450–1.73)	0.720
CPP (mm Hg)	0.950 (0.896–1.01)	0.0712
PRx	***103 (5.31–2240)***	***0.00249***
PAx	***334 (3.25–43,500)***	***0.0156***
SDNN (ms)	1.00 (0.989–1.02)	0.727
RMSSD (ms)	1.00 (0.974–1.03)	0.921
BRS (ms/mm Hg)	1.10 (0.941–1.30)	0.231
HR MSE-Ci	0.996 (0.914–1.09)	0.926
MAP MSE-Ci	***0.732 (0.611–0.867)***	***0.000409***
ICP MSE-Ci	***0.726 (0.607–0.856)***	***0.000232***
AMP MSE-Ci	***0.845 (0.748–0.948)***	***0.00510***

Regression analyses were all adjusted for age (years), Glasgow Coma Scale motor score (scale) pre-DC, infarct volume (cm^3^), and time proportion of NIC time spent on mechanical ventilation (%). Odds ratios are presented per 1 unit. *p*-Value below 0.05 was considered statistically significant. *HR* heart rate, *BPM* beats per min, *MAP* mean arterial pressure, *ICP* = intracranial pressure, *AMP* pulse amplitude of ICP, *CPP* cerebral perfusion pressure, *PRx* pressure reactivity index, *PAx* pulse amplitude index, *mechanical ventilation (%)* percentage of post-DC NIC time spent on mechanical ventilation, *SDNN* standard deviation of all normal-to-normal RR intervals, *ms* milliseconds, *RMSSD* root mean square of adjacent RR interval differences, *BRS* sequence baroreflex sensitivity, *mm Hg* millimeters of mercury, *HR MSE-Ci* multiscale entropy complexity index of heart rate, *MAP MSE-Ci* multiscale entropy complexity index of mean arterial pressure, *ICP MSE-Ci* multiscale entropy complexity index of intracranial pressure, *AMP MSE-Ci* multiscale entropy complexity index of ICP pulse amplitude

**Fig. 2:**
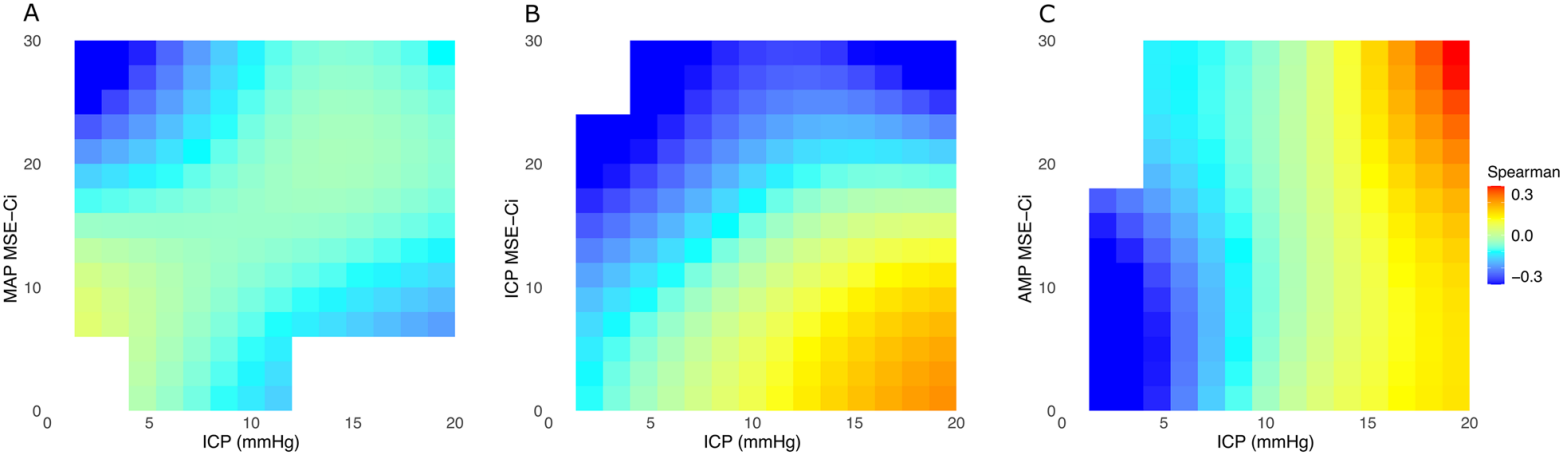
ICP vs. SC vs. mRS. Spearman correlations are done between %VMT of ICP and SC in each cell vs. mRS. Red cells denote feature combinations associated with higher mRS (worse outcome), whereas blue cells denote feature combinations associated with lower mRS (better outcome). *ICP* = intracranial pressure, *SC* signal complexity, *mRS* modified Rankin Scale, *%VMT* percentage of valid monitoring time, *MAP MSE-Ci* multiscale entropy complexity index of mean arterial pressure, *ICP MSE-Ci* multiscale entropy complexity index of intracranial pressure, *AMP MSE-Ci* multiscale entropy complexity index of ICP pulse amplitude

**Fig. 3:**
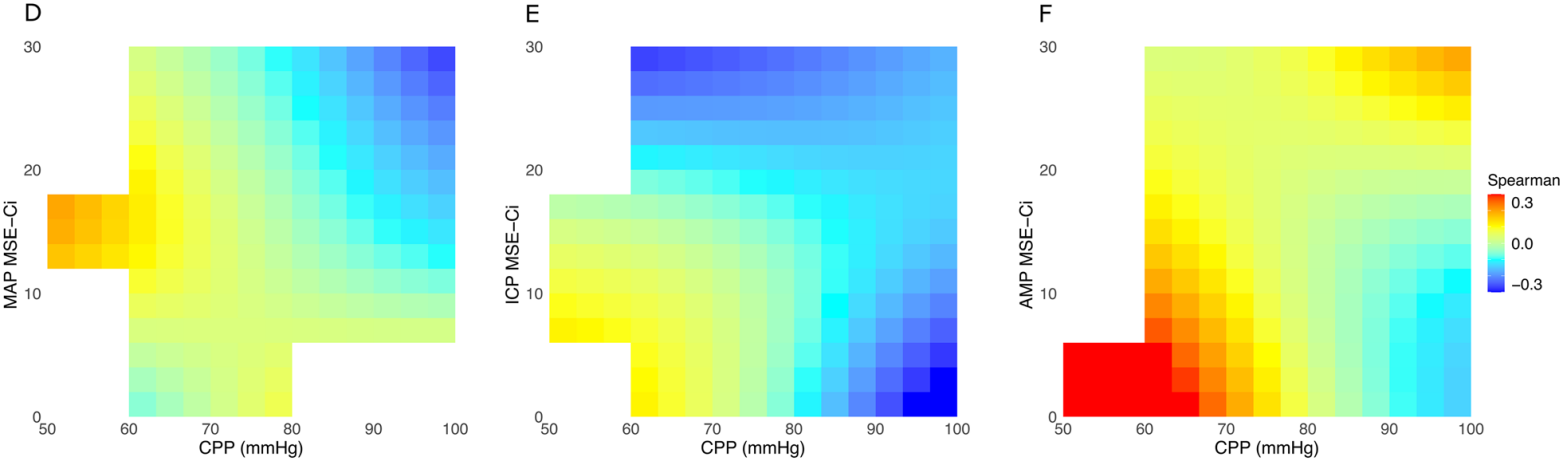
CPP vs. SC vs. mRS. Spearman correlations are done between %VMT of CPP and SC in each cell vs. mRS. Red cells denote feature combinations associated with higher mRS (worse outcome), whereas blue cells denote feature combinations associated with lower mRS (better outcome). *CPP* cerebral perfusion pressure, *SC* signal complexity, *mRS* modified Rankin Scale, *%VMT* percentage of valid monitoring time, *MAP MSE-Ci* multiscale entropy complexity index of mean arterial pressure, *ICP MSE-Ci* multiscale entropy complexity index of intracranial pressure, *AMP MSE-Ci* multiscale entropy complexity index of ICP pulse amplitude

**Fig. 4:**
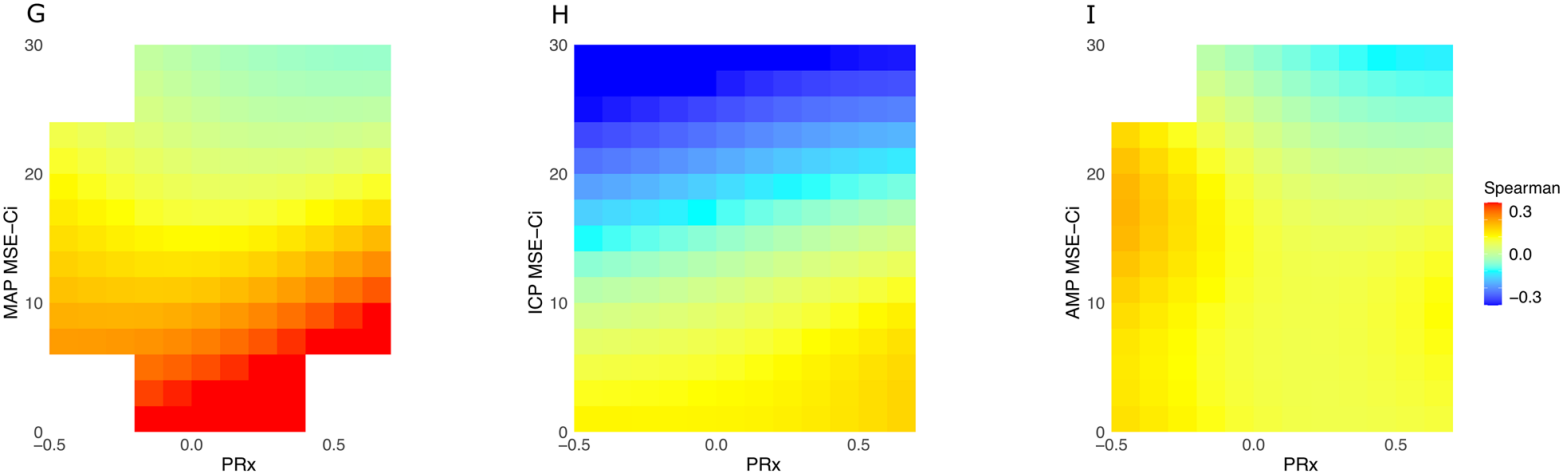
PRx vs. SC vs. mRS. Spearman correlations are done between %VMT of PRx and SC in each cell vs. mRS. Red cells denote feature combinations associated with higher mRS (worse outcome), whereas blue cells denote feature combinations associated with lower mRS (better outcome). *PRx* pressure reactivity index, *SC *signal complexity, *mRS* modified Rankin Scale, *%VMT* percentage of valid monitoring time, *MAP MSE-Ci* multiscale entropy complexity index of mean arterial pressure, *ICP MSE-Ci* multiscale entropy complexity index of intracranial pressure, *AMP MSE-Ci* multiscale entropy complexity index of ICP pulse amplitude

The interaction between these SC variables and key NIC targets were also evaluated in relation to outcome in two-variable heatmaps (Figs. 2, 3, 4). These figures showed that the combination of elevated ICP with low SC of ICP was particularly associated with worse outcome (high mRS) than such disturbances in isolation (Fig. 2B). Similarly, patients with greater burden of low CPP and low SC of MAP, ICP, and AMP recovered worse (Fig. 3A–C). Particularly combined with higher PRx, low SC of MAP and ICP seemed to be associated with worse functional recovery (Fig. 4A, B).

## Discussion

This is the first study addressing HRV, BRS, and SC on the basis of multimodal high-frequency monitoring during NIC following DC due to MMI. Consistent with reported findings of other brain injuries, e.g. TBI, ischemic, and hemorrhagic stroke [10–16, 23–26, 28–33], we observed impaired ANS including reduced HRV and BRS, as well as disturbed signal complexities of HR, MAP, ICP, and AMP in patients with MMI during the first 7 days of NIC after DC. Overall, low SC of HR, MAP, ICP, and AMP did not correlate with demographics or stroke severity, but were significantly associated with a greater burden of insults of key cerebral NIC variables, and reduced tolerance of such autoregulatory disturbances. Low signal complexities of MAP, ICP, and AMP were also independently associated with worse functional outcome. Therefore, SC may contain information that might aid in indicating the occurrence of and tolerance for secondary cerebral insults, including ICP, CPP, and PRx, to fine-tune NIC beyond the traditional treatment targets. Additionally, SC may be a useful tool for outcome prognosis in patients with MMI following DC.

ANS appeared to be impaired following DC in this cohort, with median SDNN of 112 ms, RMSSD of 28.5 ms, and BRS of 3.6 ms/mm Hg, i.e., lower compared with healthy individuals in previous studies [21, 22, 46]. These findings are consistent with reduced HRV and BRS in other brain injuries, such as TBI [15, 16, 47], and general acute ischemic stroke cohorts [10, 48, 49]. SDNN describes overall HRV, affected by both sympathetic and vagal modulation, while RMSSD reflects BtB variability, mainly influenced by parasympathetic tone [19]. Thus, the decreased SDNN and RMSSD together likely reflect suppressed vagal tone and overall autonomic imbalance. Similarly, BRS describes the heart rate response to spontaneous fluctuations and insults in blood pressure through vagal baroreflex control of the sinoatrial node [50]. Thus, reduced BRS also indicates disturbed parasympathetic modulation of heart rate in relation to blood pressure. SC is rather different from HRV and BRS, since absolute values cannot be compared across variables and cohorts. However, as SC of HR, MAP, ICP, and AMP demonstrated significant correlations with cerebral variables and outcome, we assumed that SC reflected disturbed cerebral physiology in some of our patients, in accordance with other acute brain injury conditions, such as aSAH and TBI [23–26, 28–33].

Multiple demographic and clinical factors may have affected autonomic function and SC in our cohort. Age and common comorbidities in patients with stroke, e.g., atherosclerosis, hypertension, heart failure, and coronary artery disease, are known to reduce HRV and BRS [21, 22, 46, 48, 49]. These factors may have partly contributed to disrupted ANS in this cohort with a median age of 57 years and typically heavy cardiovascular comorbidities. Interestingly, important markers of stroke severity, e.g., GCS M, midline shift, and infarct volume, did not correlate significantly with HRV, BRS, and SC either, except for higher midline shift pre-DC with higher SC of ICP and AMP. Still, the impaired ANS in patients with MMI, observed in this study, is likely partly caused by the widespread underlying brain injury caused by ischemia and space-occupying edema in important areas that control autonomic functions [10, 17]. Nevertheless, the lack of significant correlations between age and stroke severity vs. HRV, BRS, and SC can be explained by the homogeneity of the analyzed subgroup composed of older patients with MMI, which by definition is a severe and widespread hemispheric brain injury [1]. Although the pathophysiology cannot be fully elucidated, decreased HRV and BRS together may reflect sympathetic overdrive and vagal withdrawal due to central autonomic dysfunction caused by impaired insula or subsequent to injury to the brainstem in case of brain herniation [48, 50]. Additionally, mechanical ventilation and some medications administered in NIC, including sedatives, are known to decrease autonomic variability [18, 19, 44]. Most patients herein, particularly those with more severe underlying injuries or secondary brain injuries, spent the first 7 days of NIC time following DC sedated and mechanically ventilated. In other words, the sedatives together with the ventilatory support may have partly contributed to the suppressed HRV and BRS in our cohort, despite the lack of formal statistical association. Lower SC of ICP and AMP did correlate significantly with greater percentage of monitoring time in ventilatory support. However, it is difficult to specify whether the sedation and mechanical ventilation reduced intracranial SC or the reduced complexity impacted the chance of extubation.

In contrast to TBI and general ischemic stroke cohorts [10, 15, 16, 47–49], HRV and BRS were not associated with ICP, CPP, or PRx in our cohort. However, consistent with other acute brain injury conditions [23–26, 28–30], SC was significantly linked to key NIC variables in this study. For instance, higher SC of HR, MAP, and AMP correlated significantly with higher CPP and lower %VMT of CPP below 60 mm Hg. Likewise, AMP SC was significantly associated with lower %VMT of ICP above 20 mm Hg. The hypothetical mechanism is that lower SC may indicate reduced adaptive capacity to compensate for spontaneous fluctuations and pathological insults in autonomic regulation of heart rate and blood pressure through the baroreflex and autoregulatory modulation of cerebral blood flow and vascular tone [23–26, 28–31]. Hence, systemic and cerebral vascular responses to insults are less complex, and these rigid responses may therefore not fully compensate for pathological signal fluctuations [23–26, 28–31]. However, the causal direction of this association is rather difficult to define. On the one hand, low cerebral and systemic SC may not be able to properly compensate for the disrupted ANS with reduced BRS and HRV, leading to excessive fluctuations in blood pressure [51]. The result can, in turn, be impaired CPP and a heavier burden of cerebral insults, facilitating secondary brain injury and further development of edema [51]. On the other hand, physiological systems progressively lose their adaptive capacities when exceeding pathological thresholds [51], meaning that a certain burden of physiological insults may overload compensatory mechanisms, decreasing SC.

Furthermore, HRV and BRS did not correlate with outcome in our cohort, contrary to studies of general ischemic stroke and TBI [10, 15, 16, 47–49]. However, lower signal complexities of MAP, ICP (both *p* < 0.001), and AMP (*p* < 0.01) were significantly associated with worse outcome in multivariable ordinal logistic regression models adjusted for age (years), Glasgow Coma Scale motor score pre-DC (scale), infarct volume (cm^3^), and time proportion of the first 7 days of NIC spent on mechanical ventilation (%). These findings are consistent with TBI cohorts [23–26, 28–33], except for HR SC, which was not associated with outcome in our cohort. Additionally, low signal entropy of MAP, ICP, and AMP combined with high ICP, low CPP, and high PRx were particularly associated with poor outcome, possibly reflecting an exhaustion of adaptive physiological reserves to cope with such cerebral insults. This inability to compensate, especially during insults, could be linked to the loss of complexity and interdependent feedback loops [32]. Our findings suggest reduced SC to be a biomarker for pathological cerebral and systemic states, and thus associated with unfavorable outcome. Additionally, low SC may contribute to a greater burden of secondary insults of cerebral and systemic homeostasis and/or fail to compensate for such insults when they occur, possibly as low complexity reflects more rigid interactions between the cerebral and systemic vascular physiology. Thus, SC may potentially be a useful tool to aid in identifying patients more susceptible or less tolerant of secondary physiological insults as a means to fine-tune NIC post-DC and also in making outcome prognosis. However, confounders may have influenced our analyses. For instance, although systematic reviews report no difference between dominant and nondominant hemispheres in MMI outcome after DC [52, 53], patients with infarct lateralized to the right hemisphere demonstrated significantly better cerebral autoregulation (PRx) and 6-month mRS (*p* < 0.05) in this cohort. Therefore, our preliminary results require further exploration and prospective, multicenter validation before they can be implemented in clinical practice.

## Methodological Considerations

The main strength of this study is the unique, large cohort of post-DC patients with MMI, with high-frequency physiological NIC data and granular clinical and radiological records. Furthermore, our study analyzed HRV, BRS, and SC in an unexplored patient group. Still, the study has several limitations. First, it is limited by its retrospective observational design. Outcome was assessed retrospectively through chart review, which may introduce interrater variability. However, data were extracted by an experienced academic neurosurgeon (TSW) using a predefined protocol with strict variable definitions, and uncertain cases were preferably reported as missing. Although the cohort (*n* = 70) is large in the MMI context, it is still relatively small considering the risk of type I and II errors. Second, patients were sedated and mechanically ventilated in a median of 100% (42.9–100%) of the NIC time, which may affect autonomic function, including HRV, BRS, and SC [28, 32, 42]. Thus, sedation and mechanical ventilation present a confounder in this cohort. More detailed data about sedation and ventilatory support would have allowed for more in-depth analysis of the effect on outcome and NIC variables, particularly regarding specific sedative agents, as different agents may have varying autonomic effects. However, virtually all mechanically ventilated patients received continuous propofol sedation, and in some cases, opioid analgesia (predominantly morphine). Although those confounders are generally present in the clinical situation when the prognosis is evaluated, to minimize the confounding effect, the regression analyses exploring associations with mRS were adjusted for age (years), Glasgow Coma Scale motor score (scale) pre-DC, infarct volume (cm^3^), and proportion of NIC time spent on mechanical ventilation (%). An additional aspect that may reduce the granularity of the analyses is that variables were aggregated into per-patient medians before correlation analyses, as this does not consider temporal patterns. Third, the multivariable regression analyses exploring outcome associations were adjusted for four variables, meaning that each analysis included five independent variables in a cohort of 70 patients, which may cause instability or overfitting. Nevertheless, all regression analyses were also performed without adjustment, resulting in similar associations. Fourth, the pulse waves of ICP likely have different characteristics following DC compared with an intact cranial vault, as several factors can affect their form and amplitude, e.g., proximity to the removed bone flap [54]. Hence, the use of AMP may not reflect exact pulsatile ICP dynamics following DC. However, the SC of AMP seems to persistently convey important information after DC. Moreover, PAx was also explored to nuance the analyses of pulse amplitude indices, and seems to carry prognostic value as well in this cohort, which is consistent with TBI cohorts [41]. Fifth, analyses involving %VMT of ICP and CPP should be interpreted cautiously, since these percentages were generally low. Lastly, since this study was exploratory, with a statistically small cohort size, multiple unadjusted statistical tests were performed, but this increases the risk of type I error and warrants cautious interpretation of our findings.

## Conclusions

Impaired ANS, including reduced HRV and BRS, and disrupted SC of HR, MAP, ICP, and AMP were common in patients with MMI during the first 7 days of NIC following DC. Reduced SC appeared to correlate with a greater burden of secondary cerebral insults, particularly low CPP. In addition, lower systemic and cerebral SC (MAP, ICP, AMP) independently seemed to be associated with worse long-term outcome. Low SC combined with pathological levels of NIC variables (ICP, CPP, and PRx) were especially correlated with worse functional outcome of MMI following DC. This likely emphasizes the role of systemic and cerebral compensatory capacities, where low SC may indicate a reduced tolerance for secondary insults. Therefore, SC has the potential to convey valuable prognostic information and fine-tune NIC by identifying patients vulnerable to secondary autoregulatory insults and indicating the need for intervention. Multicenter, prospective studies are warranted to validate our findings.

## Supplementary Information

Below is the link to the electronic supplementary material.

Supplementary file1 (DOCX 36 KB)

Supplementary figure 1. Selection of study population. Supplementary figure 1 presents the inclusion and exclusion process of the study population. The number of eligible patients was 101. Patients with no neurointensive data (*n* = 11), patients with neurointensive data less than 24 h (*n* = 14), and patients with no outcome observations (*n* = 6) were excluded. Thus, the final cohort consisted of 70 patients.
Supplementary file2 (TIFF 15088 KB)

Supplementary file3 (DOCX 39 KB)

Supplementary file4 (DOCX 39 KB)

## Data Availability

Data are available upon reasonable request.

## Abbreviations

ABP: Arterial blood pressure
AMP: (ICP) pulse amplitude
ANS: Autonomic nervous system
BRS: Baroreflex sensitivity
BPM: Beats per min
BtB: Beat-to-beat
CPP: Cerebral perfusion pressure
DC: Decompressive craniectomy
ECG: Electrocardiography
GCS M: Glasgow Coma Scale, motor score
HR: Heart rate
HRV: Heart rate variability
IBI: Interbeat interval (RR interval)
ICP: Intracranial pressure
MAP: Mean arterial pressure
MCA: Middle cerebral artery
MMI: Malignant middle cerebral artery infarction
MSE: Multiscale entropy
MSE-Ci: Multiscale entropy complexity index
mRS: Modified Rankin Scale
NIC: Neurointensive care
PRx: Pressure Reactivity index
RMSSD: Root mean square of successive differences (of IBIs)
RR: R-to-R interval
SBP: Systolic blood pressure
SDNN: Standard deviation of normal-to-normal (IBI) intervals
SE: Sample entropy
SC: Signal complexity
TBI: Traumatic brain injury
%VMT: Percentage of valid monitoring time
