# Supplementary material for: Intracranial Pressure Monitoring, Heart Rate Variability, Baroreflex Sensitivity, and Signal Complexity During Neurointensive Care after Decompressive Craniectomy in Malignant Middle Cerebral Artery Infarction
Source: Neurocrit Care. 2026 Apr 7;44(3):803–15. doi: 10.1007/s12028-026-02506-2 (PMC13249785; doi:10.1007/s12028-026-02506-2)
Supplement: Supplementary file 1 — Supplementary file1 (DOCX 36 KB) [file 12028_2026_2506_MOESM1_ESM.docx]

STROBE Statement—checklist of items that should be included in reports of observational studies

|  | Item No. | Recommendation | Page  No. | Relevant text from manuscript |
| --- | --- | --- | --- | --- |
| **Title and abstract** | 1 | (*a*) Indicate the study’s design with a commonly used term in the title or the abstract | 2 | First sentence of “Methods” in the abstract |
|  |  | (*b*) Provide in the abstract an informative and balanced summary of what was done and what was found | 2 | Described in “Methods” and “Results” sections in the abstract |
| Introduction | | | |  |
| Background/rationale | 2 | Explain the scientific background and rationale for the investigation being reported | 3 | Explained in the “Introduction”. |
| Objectives | 3 | State specific objectives, including any prespecified hypotheses | 4 | Stated in the last paragraph of the “Introduction”. |
| Methods | | | |  |
| Study design | 4 | Present key elements of study design early in the paper | 5 | Presented in the “Patients and study design” of the “Materials and Methods” |
| Setting | 5 | Describe the setting, locations, and relevant dates, including periods of recruitment, exposure, follow-up, and data collection | 5–7 | Described in the “Materials and Methods” |
| Participants | 6 | (*a*) *Cohort study*—Give the eligibility criteria, and the sources and methods of selection of participants. Describe methods of follow-up  *Case-control study*—Give the eligibility criteria, and the sources and methods of case ascertainment and control selection. Give the rationale for the choice of cases and controls  *Cross-sectional study*—Give the eligibility criteria, and the sources and methods of selection of participants | 5 | This is explained in the “Patients and study design” of the “Materials and Methods” |
|  |  | (*b*) *Cohort study*—For matched studies, give matching criteria and number of exposed and unexposed  *Case-control study*—For matched studies, give matching criteria and the number of controls per case |  | This was not a matched study |
| Variables | 7 | Clearly define all outcomes, exposures, predictors, potential confounders, and effect modifiers. Give diagnostic criteria, if applicable | 6–9 | Described in “Data collection”, “Data analyses” and “Statistical analysis” of the “Materials and Methods” |
| Data sources/ measurement | 8* | For each variable of interest, give sources of data and details of methods of assessment (measurement). Describe comparability of assessment methods if there is more than one group | 5–9 | This is described in the entire “Materials and Methods” |
| Bias | 9 | Describe any efforts to address potential sources of bias |  | No conflict of interest. |
| Study size | 10 | Explain how the study size was arrived at | 5 | Explained in the “Patients and study design” of the “Materials and Methods” |

Continued on next page

| Quantitative variables | 11 | Explain how quantitative variables were handled in the analyses. If applicable, describe which groupings were chosen and why | 8 | Described in “Statistical analysis” of the “Materials and Methods” |
| --- | --- | --- | --- | --- |
| Statistical methods | 12 | (*a*) Describe all statistical methods, including those used to control for confounding | 8 | Described in “Statistical analysis” of the “Materials and Methods” |
|  |  | (*b*) Describe any methods used to examine subgroups and interactions | 8 | Described in “Statistical analysis” of the “Materials and Methods” |
|  |  | (*c*) Explain how missing data were addressed | 6–7 | Described in “Data collection” of the “Materials and Methods” |
|  |  | (*d*) *Cohort study*—If applicable, explain how loss to follow-up was addressed  *Case-control study*—If applicable, explain how matching of cases and controls was addressed  *Cross-sectional study*—If applicable, describe analytical methods taking account of sampling strategy | 5 | Explained in “Patient and study design” of the “Materials and Methods”. |
|  |  | (*e*) Describe any sensitivity analyses |  | NA |
| Results | | | | |
| Participants | 13* | (a) Report numbers of individuals at each stage of study—eg numbers potentially eligible, examined for eligibility, confirmed eligible, included in the study, completing follow-up, and analysed | 5 | This is explained in “Patients and study design” of the “Materials and Methods” |
|  |  | (b) Give reasons for non-participation at each stage |  | Not applicable |
|  |  | (c) Consider use of a flow diagram |  | Not applicable |
| Descriptive data | 14* | (a) Give characteristics of study participants (eg demographic, clinical, social) and information on exposures and potential confounders | 10 | Described in “Demographics and baseline characteristics” of the “Results” |
|  |  | (b) Indicate number of participants with missing data for each variable of interest | 5 | Explained in “Patient and study design” of the “Materials and Methods”. Otherwise, NA. |
|  |  | (c) *Cohort study*—Summarise follow-up time (eg, average and total amount) | 11 | Indicated in “Outcome” in the “Results”. |
| Outcome data | 15* | *Cohort study*—Report numbers of outcome events or summary measures over time | 10 | Described in “Demographics and baseline characteristics” of the “Results” |
|  |  | *Case-control study—*Report numbers in each exposure category, or summary measures of exposure |  |  |
|  |  | *Cross-sectional study—*Report numbers of outcome events or summary measures |  |  |
| Main results | 16 | (*a*) Give unadjusted estimates and, if applicable, confounder-adjusted estimates and their precision (eg, 95% confidence interval). Make clear which confounders were adjusted for and why they were included | 10–11 | This is described in the “Results” |
|  |  | (*b*) Report category boundaries when continuous variables were categorized | 6 | Described in “Data collection” of the “Materials and methods” |
|  |  | (*c*) If relevant, consider translating estimates of relative risk into absolute risk for a meaningful time period |  | NA. |

Continued on next page

| Other analyses | 17 | Report other analyses done—eg analyses of subgroups and interactions, and sensitivity analyses |  | NA. |
| --- | --- | --- | --- | --- |
| Discussion | | | | |
| Key results | 18 | Summarise key results with reference to study objectives | 12 | Described in the first paragraph of the “Discussion” |
| Limitations | 19 | Discuss limitations of the study, taking into account sources of potential bias or imprecision. Discuss both direction and magnitude of any potential bias | 15 | Discussed in “Methodological considerations” of the “Discussion” |
| Interpretation | 20 | Give a cautious overall interpretation of results considering objectives, limitations, multiplicity of analyses, results from similar studies, and other relevant evidence | 12–15 | This is considered in the entire “Discussion” |
| Generalisability | 21 | Discuss the generalisability (external validity) of the study results | 15 | Discussed in “Methodological considerations” of the “Discussion” |
| Other information | |  | | |
| Funding | 22 | Give the source of funding and the role of the funders for the present study and, if applicable, for the original study on which the present article is based | 19 | Indicated in “Funding” |

*Give information separately for cases and controls in case-control studies and, if applicable, for exposed and unexposed groups in cohort and cross-sectional studies.

**Note:** An Explanation and Elaboration article discusses each checklist item and gives methodological background and published examples of transparent reporting. The STROBE checklist is best used in conjunction with this article (freely available on the Web sites of PLoS Medicine at http://www.plosmedicine.org/, Annals of Internal Medicine at http://www.annals.org/, and Epidemiology at http://www.epidem.com/). Information on the STROBE Initiative is available at www.strobe-statement.org.
