# Supplementary figures and images for: Intracranial Pressure Monitoring, Heart Rate Variability, Baroreflex Sensitivity, and Signal Complexity During Neurointensive Care after Decompressive Craniectomy in Malignant Middle Cerebral Artery Infarction
Source: Neurocrit Care. 2026 Apr 7;44(3):803–15. doi: 10.1007/s12028-026-02506-2 (PMC13249785; doi:10.1007/s12028-026-02506-2)

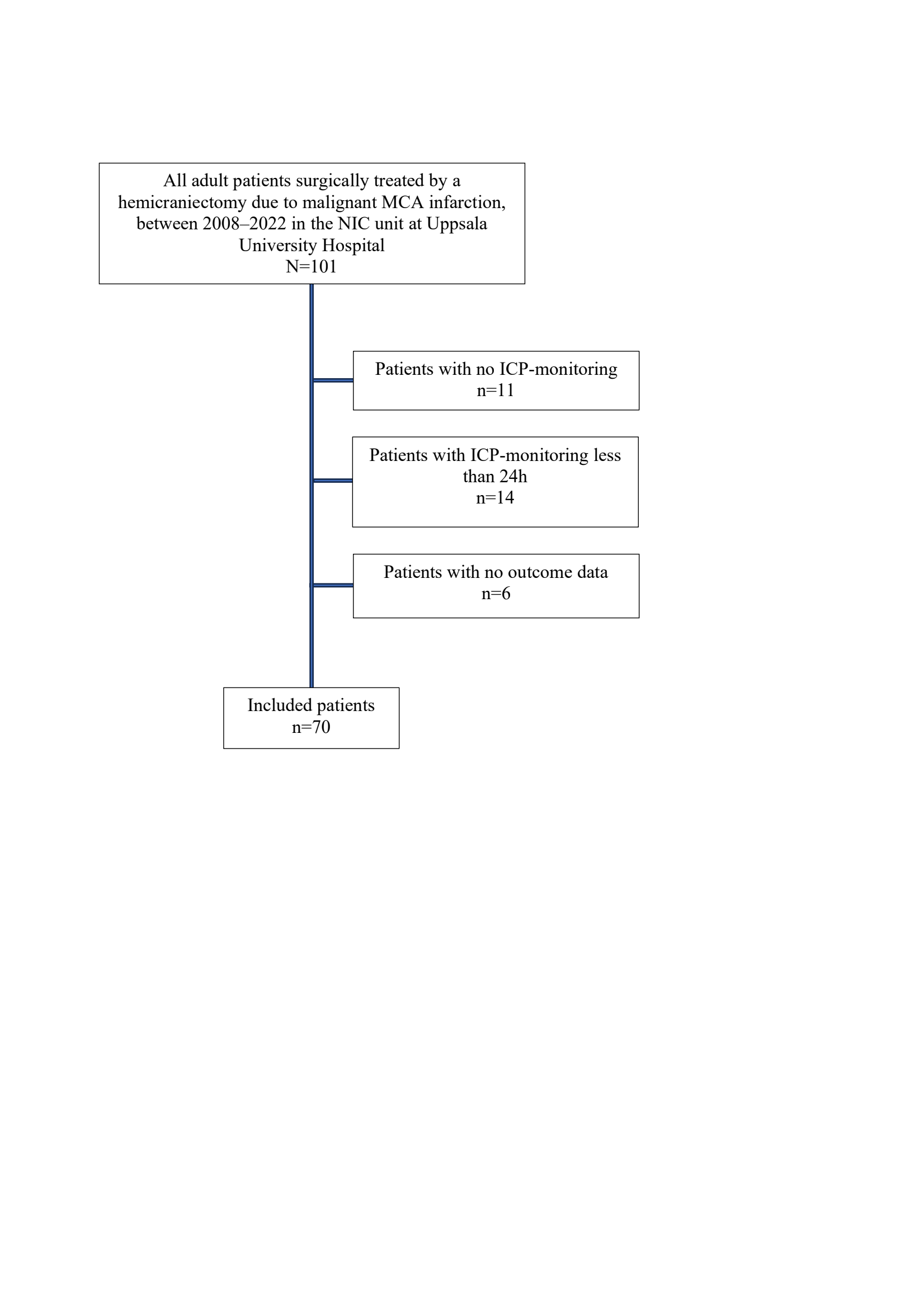

Supplement: Supplementary file 2 — Supplementary figure 1. Selection of study population. Supplementary figure 1 presents the inclusion and exclusion process of the study population. The number of eligible patients was 101. Patients with no neurointensive data (n = 11), patients with neurointensive data less than 24 h (n = 14), and patients with no outcome observations (n = 6) were excluded. Thus, the final cohort consisted of 70 patients. Supplementary file2 (TIFF 15088 KB) [file 12028_2026_2506_MOESM2_ESM.tiff]
