# Supplementary material for: Intracranial Pressure Monitoring, Heart Rate Variability, Baroreflex Sensitivity, and Signal Complexity During Neurointensive Care after Decompressive Craniectomy in Malignant Middle Cerebral Artery Infarction
Source: Neurocrit Care. 2026 Apr 7;44(3):803–15. doi: 10.1007/s12028-026-02506-2 (PMC13249785; doi:10.1007/s12028-026-02506-2)
Supplement: Supplementary file 3 — Supplementary file3 (DOCX 39 KB) [file 12028_2026_2506_MOESM3_ESM.docx]

## Supplementary table 1. Exact p-values of figure 1: Spearman’s correlation: baseline HRV, BRS, and SC vs. characteristics and cerebral variables

| Variable | SDNN (ms) | RMSSD (ms) | BRS (ms/mmHg) | HR MSE-Ci | MAP MSE-Ci | ICP MSE-Ci | AMP MSE-Ci |
| --- | --- | --- | --- | --- | --- | --- | --- |
| Age (years) | 0.675 | 0.285 | 0.695 | 0.547 | 0.0729 | 0.701 | 0.524 |
| Sex (male/female) (p-value) | 0.545 | 0.733 | 0.201 | 0.698 | 0.615 | 0.375 | 0.566 |
| GCS M before DC (scale) | 0.315 | 0.474 | 0.475 | 0.986 | 0.340 | 0.719 | 0.596 |
| Midline shift pre-DC (mm) | 0.460 | 0.582 | 0.873 | 0.519 | 0.605 | ***0.00642*** | ***0.0102*** |
| Midline shift post-DC (mm) | 0.350 | 0.515 | 0.671 | 0.956 | 0.698 | 0.782 | 0.886 |
| Infarct volume (cm^3^) | 0.754 | 0.918 | 0.270 | 0.277 | 0.498 | 0.684 | 0.0815 |
| HR (BPM) | ***0.0321*** | 0.373 | 0.903 | ***0.000207*** | 0.564 | 0.773 | 0.966 |
| MAP (mmHg) | 0.664 | 0.662 | 0.738 | 0.188 | 0.511 | ***0.0196*** | ***0.00901*** |
| Mechanical ventilation (%) | 0.241 | 0.351 | 0.178 | 0.239 | 0.109 | ***0.00632*** | ***7.70×10⁻⁶*** |
| ICP (mmHg) | 0.763 | 0.656 | 0.496 | 0.916 | 0.178 | 0.305 | 0.539 |
| VMT ICP>20 (%) | 0.439 | 0.858 | 0.891 | 0.865 | 0.0745 | 0.0875 | ***0.0420*** |
| CPP (mmHg) | 0.061 | 0.267 | 0.595 | ***0.00272*** | ***0.0327*** | 0.0576 | ***0.0401*** |
| VMT CPP<60 (%) | 0.249 | 0.331 | 0.703 | ***0.0294*** | ***0.0320*** | 0.167 | 0.161 |
| PRx | 0.310 | 0.284 | 0.465 | 0.584 | 0.431 | 0.168 | 0.597 |
| VMT PRx>0.20 (%) | 0.300 | 0.376 | 0.603 | 0.782 | 0.463 | 0.239 | 0.586 |

Significance test for sex (male/female) was done using Mann-Whitney U test. P-value below 0.05 was considered statistically significant. HRV = heart rate variability. BRS = baroreflex sensitivity. SC = signal complexity. SDNN = standard deviation of all normal-to-normal RR intervals. ms = milliseconds. RMSSD = root mean square of adjacent RR interval differences. BRS = sequence baroreflex sensitivity. mmHg = millimetres of mercury. HR MSE-Ci = multiscale entropy complexity index of heart rate. MAP MSE-Ci = multiscale entropy complexity index of mean arterial pressure. ICP MSE-Ci = multiscale entropy complexity index of intracranial pressure. AMP MSE-Ci = multiscale entropy complexity index of ICP pulse amplitude. GCS M = Glasgow Coma Scale motor score. HR = heart rate. BPM = beat per minute. MAP = mean arterial pressure. Mechanical ventilation (%) = percentage of post-DC NIC time spent in mechanical ventilation. ICP = intracanal pressure. ICP>20 (%) = %VMT of ICP above 20 mmHg. VMT = valid monitoring time. CPP = cerebral perfusion pressure. CPP<60 (%) = %VMT of CPP below 60. PRx = pressure reactivity index. PRx>0.20 (%) = %VMT of PRx above 0.20.
