# Supplementary material for: Intracranial Pressure Monitoring, Heart Rate Variability, Baroreflex Sensitivity, and Signal Complexity During Neurointensive Care after Decompressive Craniectomy in Malignant Middle Cerebral Artery Infarction
Source: Neurocrit Care. 2026 Apr 7;44(3):803–15. doi: 10.1007/s12028-026-02506-2 (PMC13249785; doi:10.1007/s12028-026-02506-2)
Supplement: Supplementary file 4 — Supplementary file4 (DOCX 39 KB) [file 12028_2026_2506_MOESM4_ESM.docx]

## Supplementary table 2. Subgroup analysis of infarct laterality

| Variable | Median (IQR) or n (%) | |  |
| --- | --- | --- | --- |
|  | Right hemisphere n=41 (100%) | Left hemisphere n=29 (100%) | p-value |
| Age (years) | 59 (52–62) | 56 (49–60) | 0.170 |
| Sex (male) | 29 (71%) | 26 (90%) | 0.0573 |
| Charlson Comorbidity Index | 0 (0–0) | 0 (0–1) | 0.328 |
| GCS M (at admission) | 6 (5–6) | 6 (5–6) | 0.384 |
| Hemiparesis at admission (yes) | 41 (100%) | 29 (100%) | – |
| Dysphasia at admission (yes) | 0 (0%) | 29 (100%) | ***<0.0001*** |
| Intravenous thrombolysis (yes) | 9 (22%) | 12 (41%) | 0.0806 |
| Endovascular thrombectomy (yes) | 2 (5%) | 5 (17%) | 0.0894 |
| GCS M (before DC) | 5 (5–6) | 5 (5–5) | 0.165 |
| Infarct volume | 241.8 (181.7–275.4) | 268.9 (203.3–328.8) | 0.109 |
| Pupillary reactivity before DC (reactive/1 unreactive/2 unreactive) | 33/8/0 (80/20/0%) | 25/4/0 (86/14/0%) | 0.532 |
| Midline shift before DC (mm) | 11 (8–13) | 11 (7–13) | 0.385 |
| Midline shift after DC (mm) | 3 (1–6) | 3 (1–6) | 0.924 |
| Basal cisterns pre-DC (open/compressed/obliterated) | 2/38/1 (5/93/2%) | 4/23/2 (14/79/7%) | 0.258 |
| Basal cisterns post-DC (open/compressed/obliterated) | 35/4/2 (85/10/5%) | 24/5/0 (83/17/0%) | 0.338 |
| Time from stroke onset to DC (h) | 38 (26–65) | 48 (30–60) | 0.858 |
| DC area (cm^2^) | 102.4 (90.8–114.4) | 106.0 (94.8–122.0) | 0.418 |
| mRS | 4 (4–4) | 4 (4–5) | ***0.0245*** |
| HR (BPM) | 76 (71–85) | 78 (64–88) | 0.853 |
| MAP (mmHg) | 89 (83–98) | 90 (85–93) | 0.519 |
| ICP (mmHg) | 11 (8–14) | 11 (10–15) | 0.706 |
| CPP (mmHg) | 77 (73–85) | 79 (75–82) | 0.654 |
| PRx | 0.19 (0.11–0.33) | 0.09 (0.06–0.19) | ***0.0170*** |
| %VMT ICP > 20 mmHg | 0.4 (0.0–2.4) | 0.7 (0.1–1.3) | 0.602 |
| %VMT CPP < 60 mmHg | 1.7 (0.3–6.1) | 1.8 (0.7–3.7) | 0.981 |
| %VMT PRx > 0.20 | 49.1 (39.6–66.4) | 39.1 (35.0–49.4) | ***0.0188*** |
| AMP (mmHg) | 11.29 (8.05–13.49) | 11.16 (9.66–14.81) | 0.758 |
| PAx | -0.111 (-0.168–-0.0427) | -0.113 (-0.174–-0.0657) | 0.586 |
| SDNN (ms) | 115.5 (99.2–146.2) | 103.7 (81.9–130.4) | 0.138 |
| RMSSD (ms) | 32.2 (23.6–38.5) | 23.2 (16.3–38.0) | 0.0722 |
| BRS (ms/mmHg) | 3.73 (2.70–6.23) | 2.50 (1.61–5.95) | 0.115 |
| HR MSE-Ci | 9.60 (6.57–15.27) | 10.50 (6.93–14.40) | 0.974 |
| MAP MSE-Ci | 14.15 (12.94–17.39) | 13.84 (11.38–15.27) | 0.0860 |
| ICP MSE-Ci | 7.70 (4.98–10.18) | 6.74 (4.82–7.80) | 0.160 |
| AMP MSE-Ci | 8.76 (5.12–15.51) | 5.66 (4.29–9.51) | ***0.0181*** |

Significance testing employed Mann-Whitney U test for continuous variables, and Chi squared test for categorical. P-value <0.05 was considered significant. GCS M = Glasgow Coma Scale motor score. DC = decompressive craniectomy. mRS = modified Rankin Scale. HR = heart rate. MAP = mean arterial pressure. ICP = intracranial pressure. CPP = cerebral perfusion pressure. PRx = pressure reactivity index. %VMT = percentage of valid monitoring time. AMP = intracranial pressure pulse amplitude. SDNN = standard deviation of normal-to-normal RR intervals. RMSSD = root mean square of successive differences of RR intervals. BRS = baroreflex sensitivity. MSE-Ci = multiscale entropy complexity index.
